# QTL mapping and analysis for drought tolerance in rice by genome-wide association study

**DOI:** 10.3389/fpls.2023.1223782

**Published:** 2023-07-25

**Authors:** Yueming Yi, Muhammad A. Hassan, Xinxin Cheng, Yiru Li, Huan Liu, Wuyun Fang, Qian Zhu, Shimei Wang

**Affiliations:** ^1^ Rice Research Institute, Anhui Academy of Agricultural Sciences, Hefei, China; ^2^ College of Agriculture, Anhui Science and Technology University, Fengyang, China; ^3^ Key Laboratory of Rice Genetics and Breeding in Anhui Province, Hefei, China

**Keywords:** rice, drought tolerance, agronomic yield traits, genome-wide association studies, quantitative trait loci (QTL)

## Abstract

Rice drought resistance is a complicated quantitative feature involving a range of biological and agronomic variables, but little is known about the underlying genetics and regulatory mechanisms that regulate drought tolerance. This study used 120 recombinant inbred lines (RILs), derived from a cross between drought tolerant Lvhan 1 and susceptible Aixian 1. The RILs were subjected to drought stress at the first ear stage, and phenotypic data of 16 agronomic and physiological traits under varying conditions were investigated. Genome-wide association study (GWAS) on the drought resistance index of traits was carried out. A total of 9 quantitative trait loci (QTLs) associated with drought-related traits were identified on chromosomes 2, 6, 7, 8, 9, and 10, which includes QTLs for plant height (PH) *qPH10.1*, effective panicles number (EPN) *qEPN6.1*, panicle length (PL) *qPL9.1*, thousand-grain weight (TGW) *qTGW2.1*, *qTGW6.1*, *qTGW8.1*, leaf length (LL) *qLL7.1*, leaf width (LW) *qLW7.1*, and leaf area (LA) *qLA7.1*. The fraction of phenotypic variation explained by individual QTL varied from 10.6% to 13.9%. Except for days to flowering (DTF), the mean values of all traits under normal water management conditions were considerably higher than those under drought conditions. Except for the DTF, the drought resistance index of all rice traits was less than 1, indicating that drought treatment reduced the EPN, FGPP, SSR, PH, and LA, which affected the growth and development of rice. The drought resistance index of DTF was 1.02, indicating that drought prolonged the heading time of rice and diminish the yield parameters. Along with identifying QTLs, the results also predicted ten candidate genes, which are directly or indirectly involved in various metabolic functioning related to drought stress. The identification of these genomic sites or QTLs that effectively respond to water scarcity will aid in the quest of understanding the drought tolerance mechanisms. This study will facilitate the marker-assisted rice breeding and handy in the breeding of drought-tolerant rice varieties.

## 1 Introduction

Rice (*Oryza sativa* L.) is an important grain crop, and water is crucial for maintaining optimal growth and development. It has been estimated that about 3000 liters of irrigation is needed to produce 1 kg of rice grains (Oladosu et al., 2019). However, in recent years, global warming, water scarcity, and frequent seasonal drought spells seriously restrict the development of China’s agriculture (Hassan et al., 2021). Rice’s development, growth, and physiological processes were severely hampered by drought (Kumar et al., 2017; Ikmal et al., 2019; Yang et al., 2021). According to Dixit et al. (2017) it is imperative to investigate the potential to improve rice productivity in facing the challenge of limited water resources. It is the need of the hour to develop rice cultivars that can withstand drought stress without compromising their yield potential (Wang & Qin, 2017). Multiple genes control rice drought tolerance. Combining rice drought tolerance gene mining with molecular marker-assisted breeding technology is beneficial for developing rice varieties resistant to drought, increasing rice yield, conserving water, and preserving national food security.

The ability of rice to withstand drought is a complicated quantitative feature regulated by numerous quantitative trait loci (QTLs). Although researchers have discovered thousands of QTLS, few have been successfully combined with breeding (Liang et al., 2021; Wang et al., 2021). Traditional QTL mapping techniques are incapable of locating genes properly and efficiently. Genome-wide association study (GWAS) has become a popular tool for breeding rice with the advancement of biotechnology. The basis of GWAS is the population’s linkage disequilibrium (LD) and single nucleotide polymorphisms (SNPs) in the genome. An analytical method that combines population structure, genome-wide LD level, and phenotypic data to identify the relationship between target traits and genetic markers/candidate genes within a population. Its advantages include high accuracy, rapid processing, and no construction of population-based mapping. GWAS provides an efficient method and approach for studying the genetic mechanism of rice drought resistance and mining potential drought resistance genes (Li et al., 2017; Bhandari et al., 2020). Wang et al. (2020) performed GWAS investigation on 272 indica materials, analyzed source-sink relationships and yield-related variables, and identified 70 QTLs influencing 11 related traits. Guo et al. (2018) used 507 diverse rice varieties to conduct a genome-wide association analysis on 51 image qualities and traditional parameters like green holding and yield, and they discovered 470 loci related to drought resistance. Additionally, employing RIL populations for GWAS and linkage analysis, 69 image trait association loci were also found. It has also been proved that some image traits and related genes can be used for drought resistance improvement in the field. Ma et al. (2016) used GWAS to find 29 QTLS related to plant height, yield, and drought resistance index in 270 cultivars. He also found a candidate gene for drought resistance, *OsRLK5*, vital in increasing rice productivity under drought stress. The GWAS based on deep sequencing is useful for detecting genetic variation in rice drought resistance enhancement. Several genes for rice drought resistance have been cloned and examined recently, including *OsMYB6* (Tang et al., 2019), *DROT1* (Sun et al., 2022), and *OsRINGzf1* (Chen et al., 2022), which have demonstrated positive benefits in controlling rice drought tolerance. However, they have not been utilized in developing new, drought-resistant rice cultivars.

In brief, GWAS has been employed to research rice attributes linked to drought resistance and to examine the genetic basis of rice drought resistance. This study constructed a recombinant inbred line population of indica rice line Lvhan 1 and drought-sensitive japonica strain Aixian 1, which had been verified in production. The population’s agronomic, physiological, and other relevant traits were examined under various water stress conditions, and GWAS was used to identify the main effect of QTLs associated with drought resistance. The results of this study provided a foundation for breeding and enhancing high-yielding and high-quality rice cultivars that are drought-resistant.

## 2 Material and methods

### 2.1 Test materials and location

The experiment was conducted in 2021 at the Lujiang Base of the Rice Research Institute of Anhui Academy of Agricultural Sciences, using the recombinant inbred line population of drought-tolerant rice Lvhan 1 and drought-sensitive rice Aixian 1, including 120 lines with significant differences in drought resistance of F_10_.

### 2.2 Test methods

#### 2.2.1 Experiment design

Conventional and drought water treatments were designed for the experiment and planted in a mobile greenhouse and an open-air field. The experiment was conducted using a randomized complete block design (RCBD). The rice seeds were sown on May 30, 2021, and transplanted on June 22, 2021. Each plot had three rows with ten plants each, with a plant and row spacing of 20 cm and 26 cm, respectively. Each water treatment was replicated three times. The open-air field is managed according to daily field management; the drought management field is 1 m higher than the ground and covered with portable greenhouses, which are managed in the open air on sunny and rainy days. The soil water potential is maintained at -15 kPa ~ 0 kPa. When the soil water potential is lower than -15 kPa, immediately supplementary irrigation is applied, and irrigation is stopped at the first ear stage of rice and subjected to drought stress. When the leaf wilting reached 50%, or the soil water potential dropped below -50 kPa for more than five days, irrigation was resumed until harvesting maturity. **Table 1** shows the weather conditions during the course of the experiment.

**Table 1:** Weather conditions during the cropping season 2020-2021.

Months	Temperature			Relative humidity	Total rainfall	Total sunshine	Mean sunshine
	Max.	Min.	Mean				
	°C	°C	°C	%	mm	h	h
May	27.1	18.7	22.9	58.0	173.5	150.4	4.9
June	30.5	22.9	26.7	59.3	100.6	146.3	4.9
July	31.5	25.3	28.4	66.3	213.1	130.1	4.2
August	30.8	24.3	27.6	68.0	354.5	131.1	4.3
September	31.1	22.3	26.7	56.9	91.2	182.9	6.1
October	21.8	13.9	17.9	59.2	73.8	125.0	4.0

Source: Meteorological station of Lujiang county, Anhui, China.

Here, mm, milli meter; d, days; h, hours.

#### 2.2.2 Investigation of agronomic traits

The following agronomic traits were investigated:

(1) Tiller number (TN): Five plants were chosen as observation points in every treatment plot. The tiller dynamics were investigated, and the effective tiller number was recorded at the heading stage.
(2) Days to flowering (DTF): The number of days from sowing to flowering was counted.
(3) Agronomic traits of leaves: After one week of drought treatment, each plot’s middle row was chosen to determine chlorophyll content (CC). The CC values in the upper, middle, and base parts of the uppermost three main stem leaves were measured by handheld chlorophyll SPAD apparatus, and the average value was calculated. Leaf length (LL), leaf width (LW), leaf area (LA), and leaf aspect ratio (LAR) were measured by the YMJ-A leaf area measuring instrument.
(4) Yield traits: Five plants were selected from each plot in each treatment at harvesting maturity to examine traits associated with yield, such as plant height (PH), effective panicles number (EPN), panicle length (PL), total grains per panicle (GPP), filled grains per panicle (FGPP), seed setting rate (SSR), thousand-grain weight (TGW), grain yield plant (GYP), and aboveground biomass (AB) which was measured by drying individual plants at 80°C till constant weight. **Supplementary File 1** shows the raw data of all above mentioned phenotypic traits.

#### 2.2.3 Calculation of drought resistance index

The drought-resistance index (DRI) was calculated using the following formula.

DRI = [measured value under drought stress/measured value under normal irrigation] ×100%.

The drought-resistance index of all traits was calculated for association analysis.

#### 2.2.4 Genotype identification

The population genotype identification was carried out by Huazhi biotechnology limited company. For this, a random leaf sample from one plant was collected for each RIL to extract DNA. Repeated PCR amplifications were performed using the Huazhi 1K rice SNP chip. The amplified fragments were obtained and constructed with a sequencing kit from Beijing Genomics Institution (BGI) and sequenced in the BGI MGISEQ-T7 sequencing machine. All experiments were conducted following the standard procedures of the sequencing kit. **Supplementary File 2** shows the raw data of genotypes in each RIL.

### 2.3 Data analysis

Excel 2018 and SPSS 26 were used for trait description and correlation analysis. The sequencing data were compared with the reference genome of Nipponbare to screen SNPs with polymorphism among parents. The correlation analysis combined the SNP marker obtained and the drought resistance index of drought resistance-related traits. The genotype data was filtered by TASSEL (v5.2.24) software first, removing the heterozygote sites, the deletion rate was less than 10%, and the genotype frequency was set to be 0.05-1.0. A total of 3550 high-quality SNP markers were selected from 5429 SNPS, and association analysis was performed using a mixed linear model(MLM)combined with Scaled IBS Kinship, genotype, and phenotypic data. The corresponding value of the observed log10p is used as the P value to plot the Q-Q scatter plot and the Manhattan plot. For the interval with a p-value less than 0.001, QTL was considered to exist in the region. The phenotypic contribution rate (R^2^) was used to evaluate the overall contribution rate of associated QTL to the drought resistance index of phenotypic traits. Refer to the reference for QTL naming principles (Yonemaru et al., 2010).

## 3 Results and analysis

### 3.1 Genetic analysis of population traits

**Figure 1:**
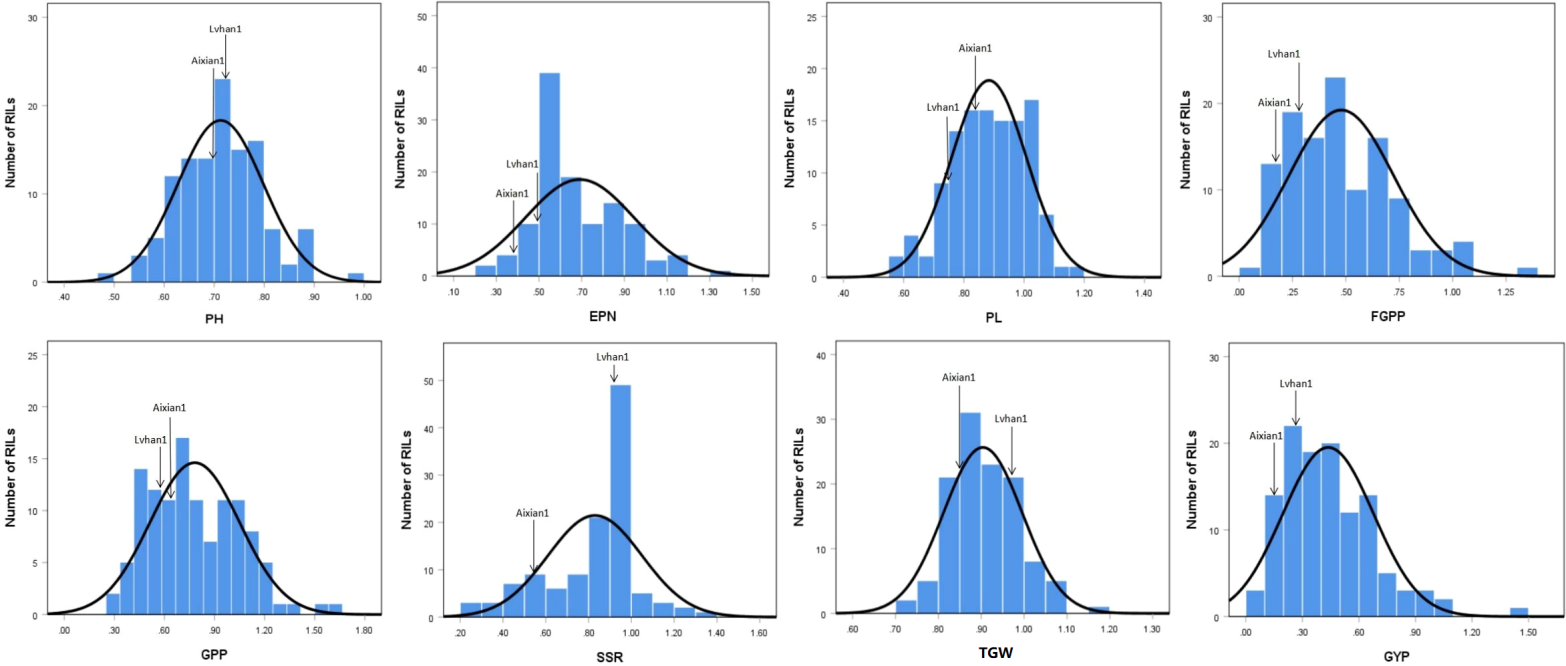
Histogram of the drought-resistance index of drought-related traits in the RIL population. PH=plant height, EPN=effective panicles number, PL=panicle length, FGPP=filled grains per panicle, GPP=grains per panicle, SSR=seed setting rate, TGW= thousand-grain weight, GYP=grain yield plant.

**Figure 2:**
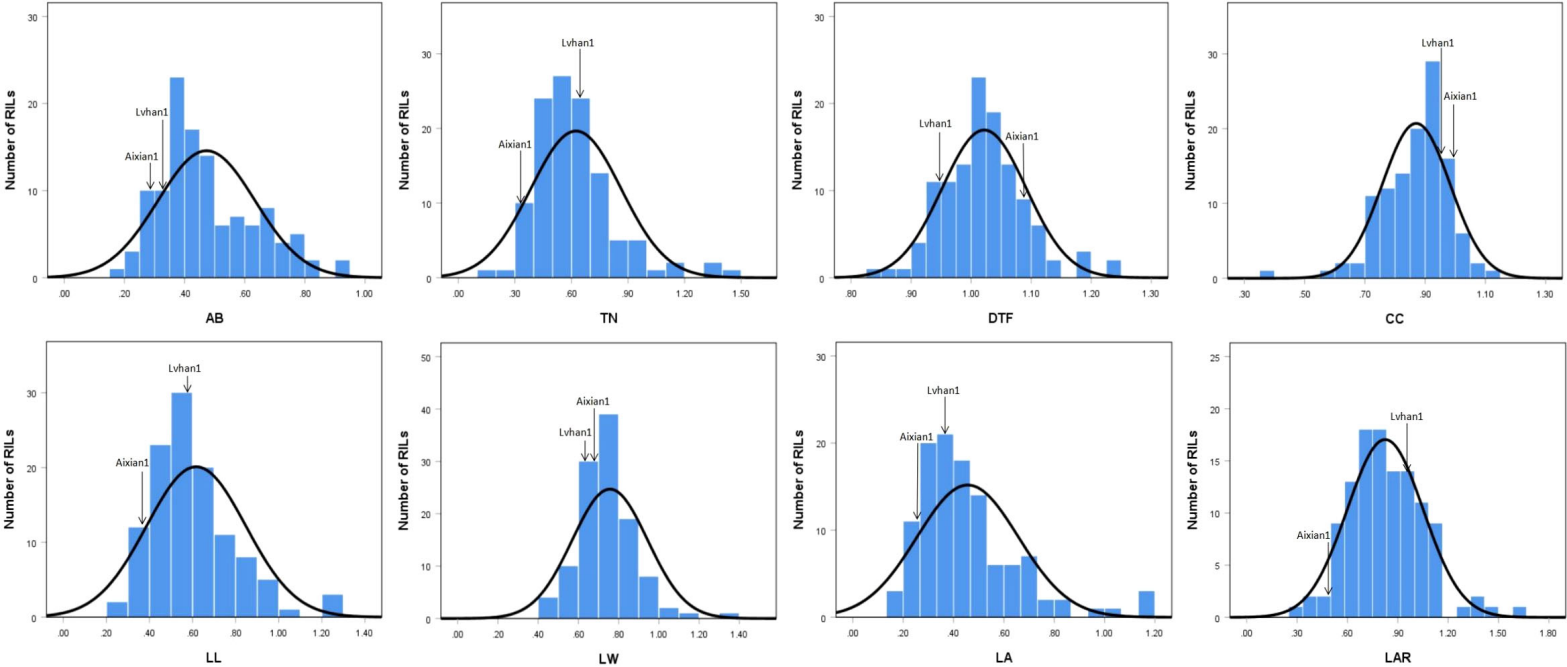
Histogram of the drought-resistance index of drought-related traits in the RIL population AB, aboveground biomass; TN, tiller number; DTF, days to flowering; CC, chlorophyll content; LL, leaf length; LW, leaf width; LA, leaf area; LAR, leaf aspect ratio.

The drought-resistance index of each agronomic character of Lvhan 1 and Aixian 1 was significantly different. The drought resistance index of each character of Aixian 1 was mostly lower than that of Lvhan 1 (**Figures 1**, **2**). Sixteen drought-resistant agronomic traits, including EPN, FGPP, SSR, PH, and LA of the RIL population, showed normal distribution, and most of the traits showed right skew and peak state. The PL is left skew, low peak state. The SSR, TGW, and CC are left skewed and in a peak state, and the data are relatively centralized. The characters with high coefficient of variation were GYP (55.18), FGPP (51.18), LA (44.17), TN (38.4), LL (37.32) and EPN (36.76). The phenotypic values of the traits showed a large range and bidirectional transgressive segregation (**Table 2**), indicating that these drought-related traits were quantitative traits controlled by multiple genes and suitable for QTL mapping. Except for the DTF, the drought resistance index of all rice traits was less than 1, indicating that drought treatment reduced the EPN, FGPP, SSR, PH, and LA, which affected the growth and development of rice. The drought resistance index of DTF was 1.02, indicating that drought prolonged the heading time of rice.

**Table 2:** Statistical analysis of the drought-resistance index of measured traits in the RIL population.

Trait	Mean value ± standard deviation	Range	Skewness	Kurtosis	Coefficient variation/%
PH	0.71 ± 0.09	0.49-0.99	0.20	0.29	12.01
EPN	0.69 ± 0.25	0.22-1.83	1.73	5.08	36.76
PL	0.88 ± 0.12	0.59-1.17	-0.25	-0.43	14.12
FGPP	0.48 ± 0.24	0.08-1.38	0.80	0.76	51.18
GPP	0.78 ± 0.27	0.31-1.6	0.50	-0.02	34.35
SSR	0.83 ± 0.22	0.21-1.31	-0.92	0.67	26.43
TGW	0.90 ± 0.09	0.42-1.2	-0.85	6.02	10.15
GYP	0.44 ± 0.24	0.07-1.5	1.14	2.33	55.18
AB	0.47 ± 0.16	0.16-0.92	0.73	-0.08	34.15
TN	0.62 ± 0.24	0.17-1.8	1.93	5.84	38.40
DTF	1.02 ± 0.07	0.85-1.24	0.47	0.90	6.85
CC	0.87 ± 0.11	0.36-1.12	-1.03	2.72	12.95
LL	0.62 ± 0.23	0.25-1.93	2.15	8.69	37.32
LW	0.76 ± 0.19	0.04-1.76	2.86	13.16	24.75
LA	0.46 ± 0.2	0.16-1.17	1.49	2.78	44.17
LAR	0.82 ± 0.23	0.29-1.61	0.56	0.91	27.43

PH, plant height; EPN, effective panicles number; PL, panicle length; FGPP, filled grains per panicle; GPP, grains per panicle; SSR, seed setting rate; TGW, thousand-grain weight; GYP, grain yield plant; AB, aboveground biomass; TN, tiller number; DTF, days to flowering; CC, chlorophyll content; LL, leaf length; LW, leaf width; LA, leaf area; LAR, leaf aspect ratio.

### 3.2 Correlation analysis

By analyzing the correlation of the drought resistance index of various agronomic and yield traits of rice, it was found that PL, FGPP, GPP, SSR, TGW, AB, CC, and PH showed a significant positive correlation (**Table 3**), indicating that various traits of rice plant affected drought resistance. A highly significant correlation existed between the number of FGPP and PH, EPN, PL, GPP, SSR, GYP, AB, and CC. The correlation coefficient between FGPP and GYP was the highest (0.981), followed by AB (0.795). The correlation between GPP and TGW achieved significant levels; the correlation coefficient was 0.211. There were highly significant correlations between GYP and yield-related traits. CC was significantly correlated with PH, PL, FGPP, GPP and SSR, and AB and DTF. There was a negative correlation between the DTF and EPN. LL was positively correlated with LW, leaf area, and LAR.

**Table 3:** The correlation of drought resistance index of measured traits.

Trait	PH(cm)	EPN	PL(cm)	FGPP	GPP	SSR	TGW(g)	GYP(g)	AB(g)	TN	DTF(d)	CC (SPAD)	LL(cm)	LW(cm)	LA (cm^2^)
EPN	-0.184														
PL(cm)	0.475^**^	-0.176													
FGPP	0.410^**^	0.382^**^	0.585^**^												
GPP	0.510^**^	-0.187^*^	0.837^**^	0.633^**^											
SSR	0.422^**^	-0.180	0.436^**^	0.565^**^	0.405^**^										
TGW(g)	0.099	0.048	0.130	0.211^*^	0.061	0.353^**^									
GYP(g)	0.413^**^	0.369^**^	0.560^**^	0.981^**^	0.599^**^	0.574^**^	0.370^**^								
AB(g)	0.248^**^	0.597^**^	0.364^**^	0.795^**^	0.457^**^	0.192^*^	0.253^**^	0.804^**^							
TN	0.075	0.211^*^	-0.022	0.077	-0.064	-0.080	0.051	0.086	0.145						
DTF(d)	0.197^*^	0.299^**^	0.181	0.166	0.205^*^	.424^**^	0.081	0.167	-0.065	-0.061					
CC(SPAD)	0.319^**^	-0.141	0.440^**^	0.299^**^	0.416^**^	0.288^**^	0.025	0.281^**^	0.196^*^	-0.102	0.221^*^				
LL(cm)	-0.040	-0.112	-0.128	-0.096	-0.065	-0.009	-0.059	-0.098	-0.080	-0.068	0.157	-0.051			
LW(cm)	-0.141	-0.020	-0.216^*^	-0.179	-0.185	-0.058	0.169	-0.134	-0.102	-0.176	0.108	-0.133	0.523^**^		
LA(cm^2^)	-0.113	-0.075	-0.150	-0.138	-0.111	-0.052	0.011	-0.125	-0.077	-0.071	0.185^*^	-0.048	0.853^**^	0.687^**^	
LAR	0.102	-0.139	0.071	0.052	0.114	0.082	-0.177	0.015	-0.029	0.013	0.080	0.078	0.771**	-0.021	0.390**

PH, plant height; EPN, effective panicles number; PL, panicle length; FGPP, filled grains per panicle; GPP, grains per panicle; SSR, seed setting rate; TGW, thousand-grain weight; GYP, grain yield plant; AB, aboveground biomass; TN, tiller number; DTF, days to flowering; CC, chlorophyll content; LL, leaf length; LW, leaf width; LA, leaf area; LAR, leaf aspect ratio.

* significance at P < 0.05; ** significance at P < 0.01.

### 3.3 Statistical analysis of SNP markers

The 5429 SNP loci were obtained by sequencing, and 3550 SNP loci were obtained by screening according to the deletion rate of less than 10% for GWAS analysis. SNP markers were evenly distributed on rice chromosomes, and the total length of the genome was 364.86 Mb, among which chromosome 1 was the longest (42.3 Mb) and chromosome 9 was the shortest (21.82 Mb). The distribution information of SNP markers on chromosomes of the whole genome is shown in **Table 4**.

**Table 4:** Distribution of SNP markers on chromosomes.

Chr	Start(bp)	End(bp)	Size(Mb)	Count	Spacing
1	222246	42526927	42.3	423	106026.77
2	1880	35926165	35.92	354	105659.66
3	398768	36102121	35.7	348	107217.28
4	332258	35126387	34.79	305	135914.57
5	170536	29536776	29.37	333	91199.50
6	122412	31198224	31.08	392	82868.83
7	16195	29564900	29.55	288	109035.81
8	17661	27999202	27.98	233	123812.13
9	465587	22289880	21.82	262	84590.28
10	46485	23155278	23.11	233	103626.87
11	1739463	28013085	26.27	332	80841.91
12	343927	27299780	26.96	238	121422.76
Total			364.86	3741	

### 3.4 Genome-wide association study

**Figure 3:**
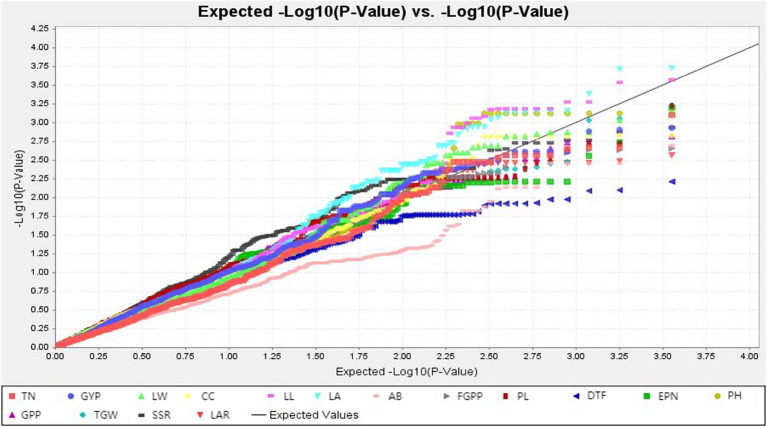
Q-Q plots illustration of drought tolerance index of measured trait. The abscissa of the Manhattan plot is 12 chromosomes of rice, the ordinate is -log10 (p) of SNPs, and the dashed horizontal line is the threshold of genome-wide significance.

The TASSEL software was used to conduct a genome-wide association study on the drought-resistance index of 16 traits in the RIL population and the selected marker loci. As can be seen from the Q-Q diagram in **Figure 3**, when the abscissa was greater than 1.5, there was a significant difference between the P value of the GWAS result and the theoretical P value, indicating that there was indeed a significant correlation between phenotype and genotype. The Q-Q diagram illustrates that the model observations are close to the anticipated values.

**Figure 4:**
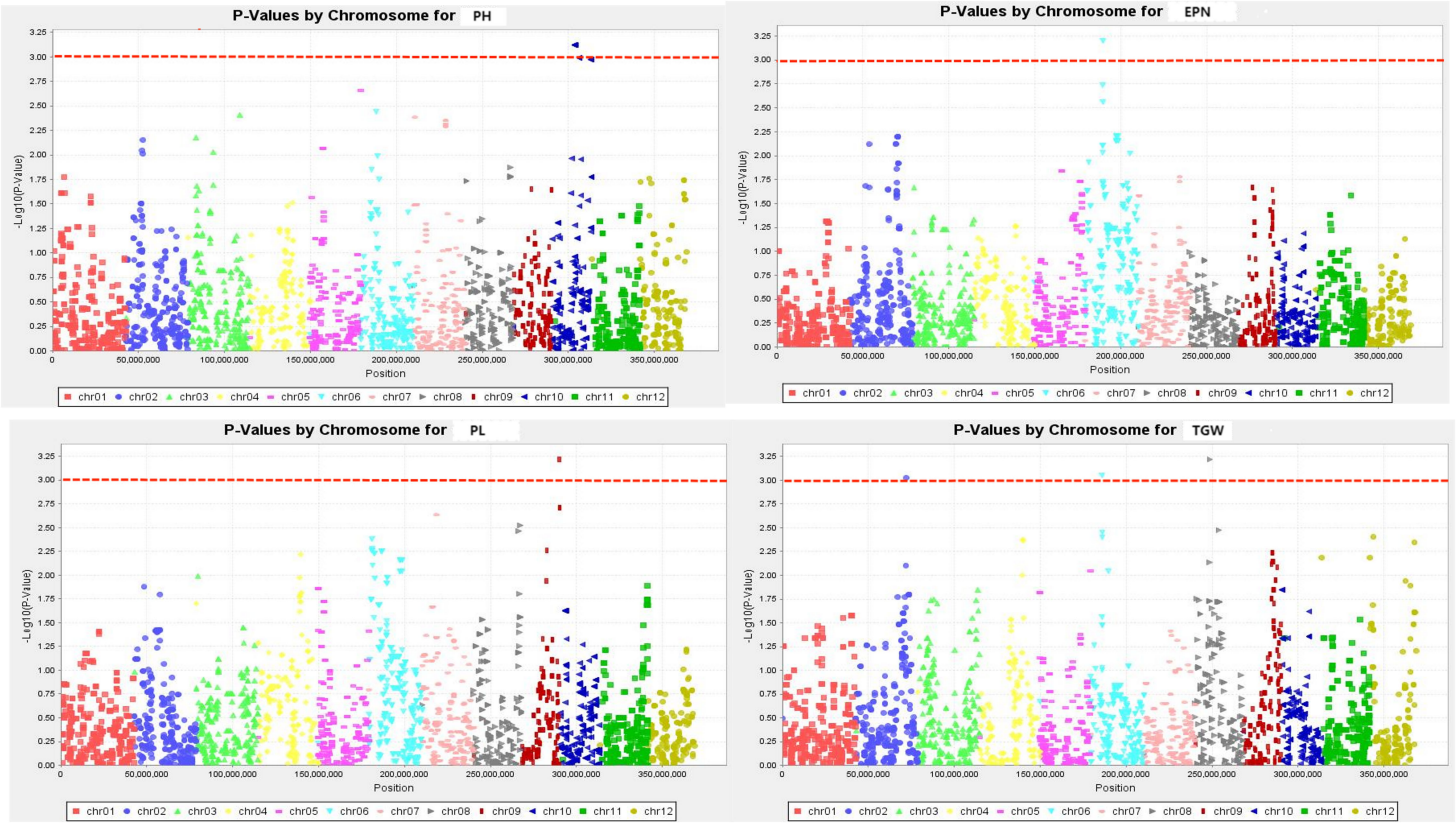
Manhattan plots exhibiting the drought tolerance index of measured traits. The abscissa of the Manhattan plot is 12 chromosomes of rice, the ordinate is -log10 (p) of SNPs, & the dashed horizontal line is the threshold of genome-wide significance.

**Figure 5:**
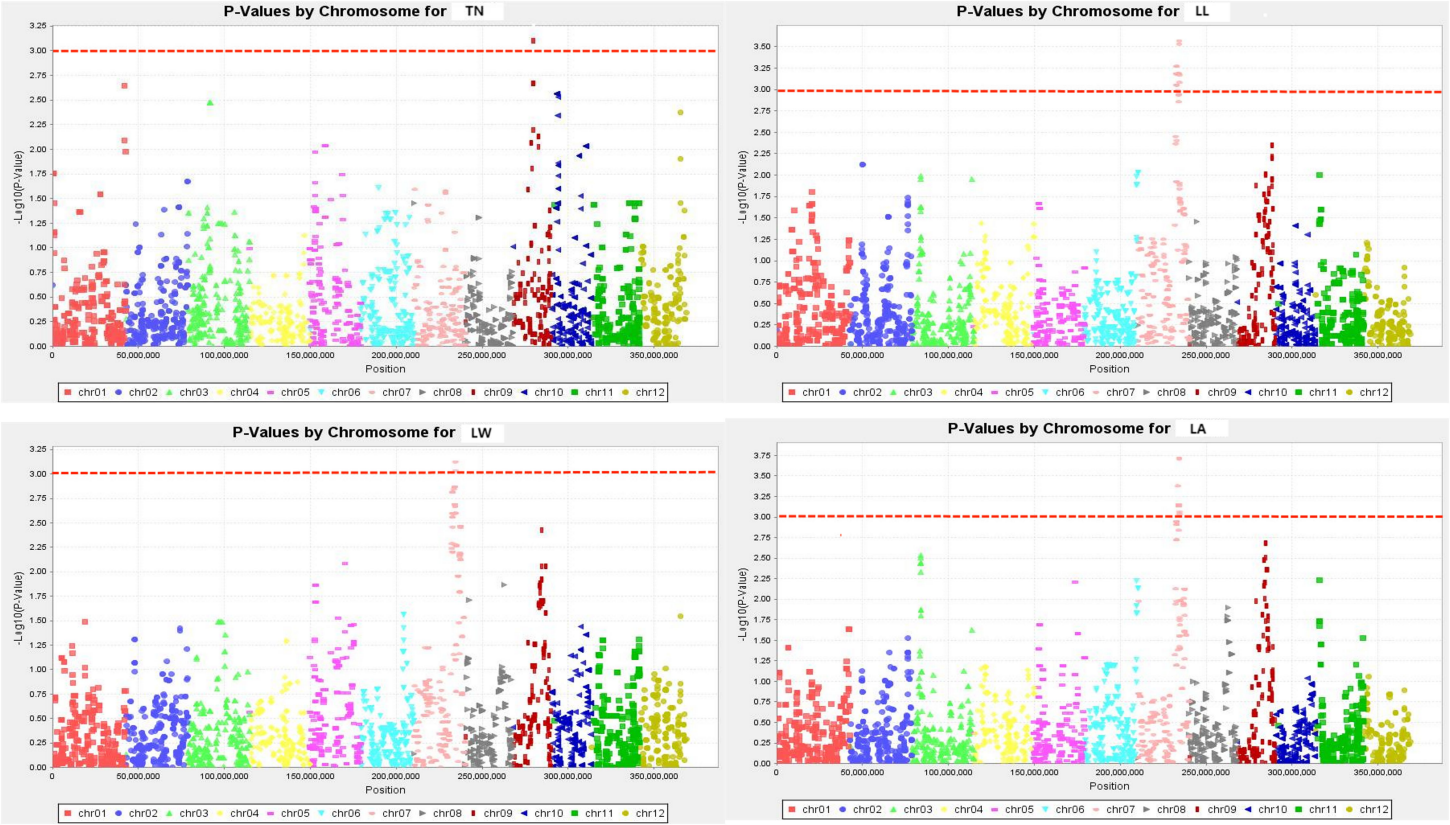
Manhattan plots exhibiting the drought tolerance index of measured traits. The abscissa of the Manhattan plot is 12 chromosomes of rice, the ordinate is -log10 (p) of SNPs, & the dashed horizontal line is the threshold of genome-wide significance.

The SNPs whose Manhattan map peak value exceeded the horizontal threshold of 3 may be the gene loci significantly associated with the measured phenotypic traits. There were 9 loci associated with rice drought resistance index located on multiple chromosomes (**Figures 4**, **5**). One leaf area-related QTL was located on chromosome 7, with a P value of 3.719, and the explanation rate of phenotypic variation was 13.9%. One QTL related to leaf length was located on chromosome 7, with a P value of 3.568, and the explanation rate of phenotypic variation was 13.2%. One QTL related to ear length was located on chromosome 9 with a P value of 3.217, and the explanation rate of phenotypic variation was 11.5%. A QTL related to effective panicle number was identified on chromosome 6, with a P value of 3.199, indicating an explanatory rate of 11.4% of phenotypic variation. One QTL related to leaf width was located on chromosome 7, with a P value of 3.124, and the explanation rate of phenotypic variation was 11.5%. A QTL related to plant height was identified on chromosome 10, with a P value of 3.121, and the explanation rate of phenotypic variation was 11.1%. Three QTLS related to centroid weight were located on chromosomes 2, 6, and 8, with P values of 3.028, 3.052, and 3.219, respectively, among which *qTGW8.1* contributed the most to the phenotype (11.6%). Detailed data are shown in **Table 5**.

**Table 5:** QTL for drought-related traits detected by association analysis.

Trait	QTL	SNP Marker	Chromosome	Position	Internal	Degrees of freedom	P*-*value	Phenotypic contribution rate R^2^(%)
PH	*qPH10.1*	GPSOS4495	chr10	13697942	13697787-13727984	12.026	3.121	11.1
EPN	*qEPN6.1*	GPSOS2698	chr06	10377542	10377542	12.394	3.199	11.4
PL	*qPL9.1*	GPSOS4239	chr09	22081469	22081469	12.488	3.217	11.5
TGW	*qTGW2.1*	GPSOS0981	chr02	29489095	29489095	11.575	3.028	10.6
TGW	*qTGW6.1*	GPSOS2563	chr06	6751340	6751340	11.693	3.052	10.7
TGW	*qTGW8.1*	GPSOS3648	chr08	8762649	8762649	12.505	3.219	11.6
LL	*qLL7.1*	GPSOS3462	chr07	23850719	22318310-24092102	14.193	3.568	13.2
LW	*qLW7.1*	GPSOS3467	chr07	24092102	24092079-24092102	12.059	3.124	11.5
LA	*qLA7.1*	GPSOS3462	chr07	23850719	23087748-24092102	14.935	3.719	13.9

### 3.5 Candidate gene association analysis

A total of 10 candidate gene loci were predicted on 12 rice chromosomes (**Table 6**). There were 23 candidate genes in the interval of 29.42 to 29.56Mb on chromosome 2. *LOC_Os02g0712000* encoding a serine endopeptidase. There were 36 candidate genes between 6.7 and 10.4Mb on chromosome 6. *LOC_Os06g0229800* was a cloned *ALK*, a key gene controlling rice’s gelatinization temperature and encoding soluble starch synthase II (Raza et al., 2020). *LOC_Os06g0286500* encodes *NBS-LRR* disease resistance protein homologous; There were 261 candidate genes between 22.2-24.2MB on chromosome 7, among which *LOC_Os07g05868* encoded a triacylglycerol lipase. *LOC_Os07g0586700* encodes the transcriptional suppressant *HOTR*. *LOC_Os07g0571800* is similar to the *YABBY4* gene, which regulates plant growth and development by regulating the gibberellin pathway (Yang et al., 2016). *LOC_Os07g0558500* encodes a fibro-alcohol tyrosine phosphatase antibody protein closely related to the *NYC4* gene, which is involved in the degradation of chlorophyll-protein complexes during leaf aging (Yamatani et al., 2013). *LOC_Os07g0558400* encodes chlorophyll a-b binding protein *CP29.1*, and *LOC_Os07g0591100* encodes *DUF620* family proteins. Seven candidate genes were in the 8.7-8.8MB region on chromosome 8, *LOC_Os08g0243500* encoding NADPH oxidoreductases. *LOC_Os09g0360400* encodes a holoenzyme synthase, and *LOC_Os09g0555800* encodes a protein-containing AMP binding domain. There were 29 candidate genes in the 11.7-22.2MB range of chromosome 9. Five candidate genes encoding an oxidoreductase were between 13.6 and 13.7Mb of chromosome 10, *LOC_Os09g0403400*.

**Table 6:** Candidate genes of important QTLs.

QTL	Candidate gene	Gene description
*qPH10.1*	*LOC_Os10g0403700*	oxido-reductase
*qEPN6.1*	*LOC_Os06g0286500*	*NBS-LRR* disease-resistance protein homologs
*qPL9.1*	*LOC_Os09g0555800*	Amp-binding domains containing proteins
*qTGW2.1*	*LOC_Os02g0712000*	Serine endopeptidase
*qTGW6.1*	*LOC_Os06g0229800*	*ALK* gene
*qTGW8.1*	*LOC_Os08g0243500*	NADPH oxido-reductase
*qLL7.1*	*LOC_Os07g0558400*	Chlorophyll a-b binding protein
*qLA7.1*	*LOC_Os07g0571800*	*YABBY4* gene
*qLA7.1*	*LOC_Os07g0586700*	transcription repressor *HOTR*
*qLL7.1/qLA7.1*	*LOC_Os07g0581300*	
*qLL7.1/qLW7.1/qLL7.1*	*LOC_Os07g0591100*	*DUF620* family protein

## 4 Discussion and conclusion

### 4.1 Heritability and correlation analysis of drought-resistant traits

Except for the DTF, the drought resistance index of all rice traits was less than 1. These results indicated that the phenotypic values of each trait under water stress were lower than those under normal water conditions. Drought stress had serious effects on rice morphology, yield, and physiology. Drought stress lengthened the heading time while decreased growth period and seed setting rate; this resulted in the significant reduction in the rice grain yield (Shamsudin et al., 2016; Sandhu & Kumar, 2017). The variation coefficients of FGPP and GYP were the highest under flood and drought conditions, indicating that drought had the most serious effect on grain number and yield. There was a significant positive correlation between yield-related and physiological traits in rice. The GYP was positively correlated with EPN, GPP, FGPP, and SSR, which was consistent with the analysis of Cu et al. (2021). There was a significant positive correlation among leaf traits.

### 4.2 GWAS location analysis

A total of 9 QTL loci related to drought resistance traits were identified by GWAS, which was distributed on chromosomes 2, 6, 7, 8, 9, and 10, which may provide important genetic resources for future breeding. In order of contribution rate value, they are *qLA7.1*, *qLL7.1*, *qTGW8.1*, *qPL9.1*, *qEPN6.1*, *qLW7.1*, *qPH10.1*, *qTGW6.1*, *qTGW2.1*. The site with the largest contribution rate is *qLA7.1*, which is related to leaf area. Studies have concluded that leaves are one of the most sensitive organs in plants to drought stress, and leaf area is negatively correlated with drought tolerance. Under mild drought stress, biomass accumulation was inhibited, plant area decreased, and leaf number decreased. Under severe drought stress, plant leaves will age, die and fall off rapidly (Chen et al., 2023).

The QTL related to plant height was located on chromosome 10. The QTL related to effective panicles number was located on chromosome 6. The physical location of the loci related to panicle length located by Zheng et al. (2022) on chromosome 9 was similar to the *qPL9.1* loci region located in this study. QTLS related to 1000-grain weight were located on chromosomes 2, 6, and 8. The QTL related to the tiller number was located on chromosome 9. QTLS related to leaf length, leaf width, and leaf area were located on chromosome 7, and the loci of *qLL7.1* and *qLA7.1* overlap. The two loci are on the same chromosome, and there may be gene linkage. The phenotypic contribution rate of QTL loci ranged from 13.2% to 13.9%. Zhu and Xiong (2013) studied 1016 global rice core germplasm resources using GWAS analysis and identified 12 QTLS affecting the length of the rice blade, 7 QTLS affecting the width of the rice blade, and 6 QTLS affecting the area of the rice blade. The loci related to yield and leaf traits of each plant in this study were not similar to those of Zhu and Xiong (2013) so they may be new loci.

Leaf length, leaf width, and leaf area were controlled by *LOC_Os07g0591100* closely linked gene, and leaf length and leaf area were controlled by *LOC_Os07g0581300* closely linked gene, which suggested that there was a polytropic between the two genes. *LOC_Os02g0712000*, *LOC_Os06g0229800*, and *LOC_Os08g0243500* tightly linked genes control the 1000-grain weight. *LOC_Os02g0712000* encodes a serine endopeptidase. *LOC_Os06g0229800* is an *ALK* gene. *LOC_Os08g0243500* encodes a NADPH oxido-reductase. *Loc_os08g0243500* is a crucial gene of rice gelatinization temperature, promoting starch and sucrose metabolism. The panicle length was mainly controlled by *LOC_Os09g0555800* and *LOC_Os09g0555850*, closely linked genes. *LOC_Os09g0555800* encodes a protein-containing AMP binding domain, and *LOC_Os09g0555850* does not predict a known function. Effective panicle number was controlled by *LOC_Os06g0286500* closely linked gene *LOC_Os06g0286500* encoding *NBS-LRR* resistance protein homolog; Tiller number was mainly controlled by *LOC_Os09g0360400* closely linked gene, encoding a holoenzyme synthase. Plant height was controlled by LOC_Os10g0403400 and *LOC_Os10g0403700* closely linked genes. *LOC_Os09g0403400* encodes an oxidoreductase, and *LOC_Os10g0403700* has no predictive function.

### 4.3 Conclusion

Breeding for abiotic conditions like drought remains challenging due to the complex nature of the genetic system and the risk of unexpected developments (Debnath et al., 2023). To find the genes that control characteristics of drought tolerance, a modern molecular technique called QTL analysis was employed. In this study, nine QTL loci related to drought resistance were identified and located on chromosomes 2, 6, 7, 8, 9, and 10, which includes *qPH10.1*, *qEPN6.1*, *qPL9.1*, *qTGW2.1*, *qTGW6.1*, *qTGW8.1 and qLL7.1*, *qLW7.1* and *qLA7.1*, respectively. The higher contribution rate sites were *qLA7.1* related to leaf area, and *qLL7.1*, related to leaf length. Ten candidate genes were also predicted, that includes *LOC_Os10g0403700* (oxido-reductase), *LOC_Os06g0286500* (*NBS-LRR* disease-resistance protein homologs), *LOC_Os09g0555800* (Amp-binding domains containing proteins), *LOC_Os02g0712000* (Serine endopeptidase), *LOC_Os06g0229800* (*ALK* gene), *LOC_Os08g0243500* (*NADPH* oxido-reductase), *LOC_Os07g0558400* (Chlorophyll a-b binding protein), *LOC_Os07g0571800* (*YABBY4* gene), *LOC_Os07g0586700* (transcription repressor *HOTR*), and *LOC_Os07g0591100* (*DUF620* family protein). It is necessary to continue verifying and fine-locating these QTLs and candidate genes through the conduction of genetic transformation and functional verification of candidate genes. This research study laid a foundation for a better understanding of rice drought tolerance genetic basis and facilitated the rice breeding program with respect to drought tolerance.

## Data availability statement

The original contributions presented in the study are included in the article/**Supplementary Files**, further inquiries can be directed to the corresponding author/s.

## Author contributions

YY and MH designed the experiment and wrote the manuscript. XC, SW and QZ contributed to the data interpretation and writing. YL and HL designed the figures and formulated tables. WF, QZ, MH, and YY participated in the processing of the experimental material. All authors read and approved the final manuscript.
